# Changes in Digestive Enzyme Activities during Larval Development of Spotted Seatrout (*Cynoscion nebulosus*)

**DOI:** 10.1155/2024/1309390

**Published:** 2024-05-08

**Authors:** Martín Alberto Arenas-Pardo, Martha Gabriela Gaxiola-Cortés, Alvaro Fabricio Barreto-Altamirano, Adriana del Carmen Paredes-Medina, Iveth Gabriela Palomino-Albarrán, Patricia Margarita Balam-Uc, Juan Carlos Maldonado-Flores, Carlos Alfonso Álvarez-González

**Affiliations:** ^1^Laboratorio de Fisiología en Recursos Acuáticos, División Académica de Ciencias Biológicas, Universidad Juárez Autónoma de Tabasco (UJAT), 0.5 km Carretera Villahermosa-Cárdenas, 86000, Villahermosa, Tabasco, Mexico; ^2^Unidad Multidisciplinaria de Docencia e Investigación (UMDI) Sisal, Facultad de Ciencias, Universidad Nacional Autónoma de Mexico (UNAM), Puerto de Abrigo, 97356, Sisal, Yucatán, Mexico

## Abstract

The spotted seatrout (*Cynoscion nebulosus*)—an important commercial species—has a high potential for aquaculture in the Gulf of Mexico. To optimize its feeding during larval rearing, this study aims to evaluate the primary gastric (pepsin), intestinal (leucine aminopeptidase and alkaline phosphatase), and pancreatic (alkaline protease, trypsin, chymotrypsin, amylase, and lipase) enzyme activities from hatching to day 30. A multivariate analysis identified three digestive enzyme development stages during the spotted seatrout larval transformation. The first stage occurred between 1 (mean ± standard error (SE) = 1.73 ± 0.14 millimeter (mm) standard length (SL)) and 3 (2.14 ± 0.07 mm SL) days after hatching (DAH); a period of digestive stability showed the highest activity in amylase and bile salt-dependent lipase. The second stage (from 4 (2.53 ± 0.09 mm SL) to 20 (10.92 ± 0.51 mm SL) DAH) was a period of digestive transition, during which leucine aminopeptidase, chymotrypsin, and alkaline proteases were identified as the predominant enzymes from 4 to 5 DAH. In the third stage—a period of digestive stability—pepsin was the major enzyme that occurred between 25 (16.51 ± 0.81 mm SL) and 30 (25.91 ± 0.82 mm SL) DAH. These results indicate that the spotted seatrout larvae have a digestive system adapted to lipids and carbohydrates at the onset of feeding, with an immediate transition to protein digestion when exogenous feeding begins. Additionally, the digestive system of the spotted seatrout may be considered mature at 25 DAH. Further research is needed to elucidate the mechanisms of digestive tract development in the spotted seatrout larvae.

## 1. Introduction

The larval stage is one of the most critical factors in marine fish culture. Fish larvae necessitate specific biotic and abiotic conditions to ensure their survival, proper development, and growth [1]. One of the major bottlenecks in larval fish culture is the high mortality during the transition from endogenous to exogenous feeding and the weaning period [2]. During the transition from larva to juvenile, the digestive tract undergoes major anatomical and functional changes from a short and straight tube (immature) to a segmented and histologically differentiated tract (mature) [3]. Simultaneously, there exists a sequential chronology in the variation of gastric, pancreatic, and intestinal enzymes; nevertheless, the timing of these processes varies among species, dependent on their life histories [4]. Knowledge of the development of digestive enzymes during the transformation from larvae to juveniles provides information on the digestive process that can be synchronized with feeding and rearing protocols [5]. In this sense, numerous studies on ontogenetic changes in the digestive enzymes of fish larvae have been conducted to understand their digestive physiology [6–12]. Identifying physiological biomarkers of the digestive system in fish larvae has allowed the appropriate design of compound diets and pertinent weaning during larviculture [5, 13].

The spotted seatrout (*Cynoscion nebulosus*, Sciaenidae) is a carnivorous marine fish distributed along the Western and Northwestern Atlantic coasts [14]. This species is of high value for commercial and recreational fishing in Mexico and the United States of America [15, 16]. The spotted seatrout is a euryhaline [17], eurythermal [18], fast-growing species with a low food conversion rate [19, 20]; its reproduction is possible in captivity [21], a characteristic that makes it suitable for aquaculture [22]. In this context, protocols have been developed to induce reproduction and spawning in captivity for the spotted seatrout, both naturally and by using exogenous hormone therapies [23–25]. However, to our knowledge, no studies have been available on the ontogenetic development of the digestive system in the spotted seatrout larvae. Thus, the present study aims to evaluate the development of digestive enzyme activity (stomach, pancreas, and intestine) in spotted seatrout larvae to provide basic knowledge on digestive physiology to optimize their feeding protocols and weaning processes.

## 2. Materials and Methods

### 2.1. Egg Production and Larviculture

Wild adults of the spotted seatrout were captured at Sisal Beach, Yucatán, during their breeding season in May 2023 and transported to the Unidad Multidisciplinaria de Docencia e Investigación (UMDI) Sisal, Universidad Nacional Autónoma de México (UNAM). Seven females (606.8 ± 137 g (mean ± standard deviation (SD)), capable of spawning according to Lowerre-Barbieri et al. [26], and three males (463 ± 26.9 g), exhibiting the presence of milt upon light abdominal pressure, were induced to spawn with a single injection (1,100 UI kg^−1^ body weight) of human chorionic gonadotropin (CG5, SIGMA) [21]. The broodstock was placed in an 18-m^3^ cylindrical fiberglass tank (2 m in diameter, 1.5 m in height) under natural temperature and photoperiod (outdoor), utilizing a continuous water flow system and aeration. Spontaneous spawning occurred after 37 hr of hormonal induction. The eggs were collected in a 200-L capacity cylindrical fiberglass tank equipped with 0.5-mm mesh screens, positioned under the outflow of the spawning tank.

The viability of the collected eggs was assessed by recording the percentage of floating eggs. To estimate the total number of fertilized eggs, three 0.1-mL samples of floating eggs were counted, and the mean value of these samples was extrapolated to the total volume of floating eggs. Additionally, the mean diameter of the fertilized eggs was calculated by measuring three samples, each consisting of 100 fertilized eggs. Larval rearing was conducted in three cylindrical fiberglass tanks, each with a capacity of 450 L (100 cm in diameter, 58 cm in height). The initial stocking density was 95 fertilized eggs per liter, with 734.5 ± 0.0 *µ*m in diameter. Hatching occurred 15 hr after incubation, and the eggs that did not hatch were removed with a siphon to avoid contamination by organic matter decomposition. The larval stocking density after hatching was 93 organisms per liter. The larval stock density after hatching was estimated by incubating three samples, each containing 100 fertilized eggs, in 2-L beakers.

**Figure 1:**
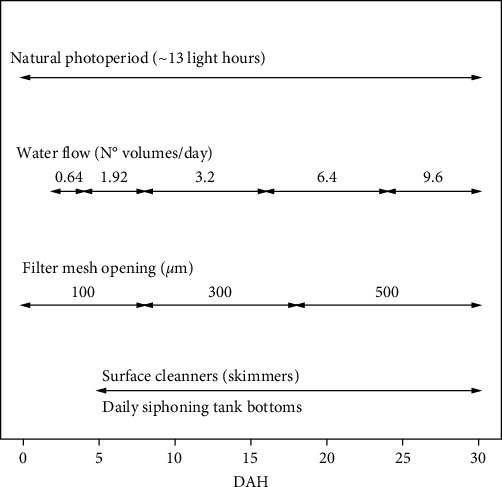
Water quality and environmental management protocols during larviculture of spotted seatrout (*C. nebulosus*).

A water quality and environmental management protocol for egg incubation and larval rearing is shown in Figure 1. The seawater used for larval rearing was treated in a pressurized sand filter (SD70, Pentair Inc., MN, USA), a multicartridge filter (P005 and P010, Filter Specialists, Inc., MI, USA), and an ultraviolet–visible (UV) lamp (R-pkbal.plat30, Purikor, NL, Mexico). Water quality during egg incubation and larval rearing were salinity (35 g/L), dissolved oxygen (4.8 ± 0.1 mg/L), temperature (28.9 ± 0.8°C), pH (7.8 ± 0.0), ammonia (0.3 ± 0.1 mg/L), nitrites (0 mg/L), and nitrates (7.2 ± 5.0 mg/L).

Table 1 shows the feeding regime used during the rearing period. The yolk was absorbed 15–24 hr after hatching, and then the larvae were fed four times a day (6:00, 10:00, 14:00, and 18:00 hr). The feeding regime was based on live food and commercial feeds; larvae were fed with rotifers (*Brachionus rotundiformis*) from 2 to 15 days after hatching (DAH) and nauplii and metanauplii of *Artemia franciscana* Biogrow® (Proveedora de Insumos Acuícolas, S. A. de C.V. Mazatlán, SIN, MX; protein 70.2%, lipid 20.8%, and ash 6.1%) from 9 to 17 and 16–30 DAH, respectively. Rotifers were enriched with microalgae (*Nannochloropsis oculata*) and Origreen (Skretting, Stavanger, Norway; protein 43%, lipid 30%, and ash 12%), whereas *Artemia* metanauplii were enriched with Selco® (INVE Aquaculture, Belgium). Dry-formulated food Otohime® (Marubeni Nisshin Feed Co., Ltd., Japan; protein 56.3%, lipid 15.9%, ash 13.5%, and particle size 75–1,410 *μ*m) was provided to larvae from 9 to 30 DAH.

### 2.2. Fish Sampling

According to Blaylock et al. [22], the larval rearing period of spotted seatrout was 30 days after hatching. During larval rearing, a variable number of larvae were collected for measurement of digestive enzyme activities. Larvae were collected 3.5 hr after the first feeding of the day (at 09:30hr) to ensure an empty digestive tract [27]. Twenty larvae were collected at 1 DAH (eleutheroembryo), and 10 larvae were collected at 3, 4, 5, 7, 10, 15, 20, 25, and 30 DAH per tank using a 250-*µ*m net. Larvae were euthanized using eugenol overdose under cold water conditions (0–4°C), rinsed with distilled water, frozen in liquid nitrogen, and stored at −80°C until analysis. The official Mexican Norm (NOM-062-ZOO-1999 from Secretaría de Agricultura, Ganadería, Desarrollo Rural, Pesca y Alimentación) was followed for animal welfare practices. The standard length (SL) of each larva was obtained by measuring five larva samples per tank from the lower jaw to the notochord with an optical microscope (VE-BC3 Plus, Velab). Larval growth was obtained from 10 pooled samples/tank using an analytical balance (Ohaus, NJ, USA; precision of 10^−4^ g). Thermal age units (cumulative degree DAH, CTU) were used to compare closely related species. The temperature units accumulated by day 0 embryos were zero; day 1 fish accumulated the mean temperature of day 0, day 2 fish accumulated the mean temperature of day 0 and day 1, and so on.

### 2.3. Biochemical Analysis

Whole tissues of embryos and larvae were homogenized in 30 volumes (v/w) [28] of ice-cold (0–4°C) universal buffer (25 mmol, pH 7) using pellet pestles and an Ultra Turrax IKA T18 homogenizer (IKA Works, Inc., NC, USA). Homogenates were centrifuged at 9,000*g*/10 min at 4°C; the precipitates were discarded, and the supernatants were again centrifuged at 30,000*g*/30 min at 4°C. The resulting supernatants were separated to measure pepsin, total alkaline proteases, trypsin, chymotrypsin, amylase, lipase, and leucine aminopeptidase. The pellets resulting were dissolved in tris-mannitol buffer (50 mmol/ L mannitol, 2 mmol/L tris; pH 7) to measure enzyme alkaline phosphatase [29].

A Biotek Synergy 2 SL microplate reader (BioTek Instruments, Inc., VT, USA) was utilized to determine the activity of the gastric, pancreatic, cytosolic, and brush border enzymes. Pepsin (EC 3.4.11.1) activity was measured using hemoglobin: HCl 0.5% as substrate, pH 2 [30]. Alkaline protease activity was determined according to Nolasco-Soria [31] using casein 0.25% as substrate in a universal buffer (50 mmol/L, pH 8). The quantity of enzyme that releases 1 *µ*mol of amino groups per minute was used to define one unit (U) of pepsin and alkaline protease activity, utilizing the *o*-phthalaldehyde (OPA)-dithiothreitol (DTT) reagent [31] and measured at 330 nm. An L-serine standard curve was used to determine the number of hydrolyzed peptide bonds.

Trypsin (EC 3.4.21.4) activity was quantified using 1 mmol/L N-benzoyl-DL-arginine-p-nitroanilide hydrochloride (BAPNA) as substrate in 50 mmol/L Tris-HCl and 10 mmol/L CaCl_2_ at pH 8 [32]. Leucine aminopeptidase (EC 3.4.11.1) was assayed using 0.1 mmol/L leucine p-nitroanilide as substrate in 50 mmol/L sodium phosphate at pH 7.2, according to Maroux et al. [33]. Chymotrypsin (EC 3.4.21.1) was determined using 0.1 mmol/L N-succinyl-ala-ala-pro-phe p-nitroanilide (SAAPNA) as substrate in 50 mmol/L Tris-HCl and 10 mmol/L CaCl_2_ at pH 7.8 [34]. One unit of enzymatic activity was defined as 1 *µ*mol p-nitroanilide released per minute and measured at 410 nm. A p-nitroanilide curve was used to determine the hydrolyzed number of micromoles of p-nitroanilide.

Alkaline phosphatase (EC 3.1.3.1) activity was quantified using as substrate 1 mmol/L 4-nitrophenyl phosphate in 50 mmol L Tris-HCl at pH 8.5 [29]. Bile salt-dependent lipase (EC 3.1.1.1) was determined according to Gjellesvik et al. [35], using as substrate 0.4 mmol L 4-nitrophenyl caprylate in 50 mmol/L Tris and 6 mmol/L sodium taurocholate at pH 7.4. One unit of enzymatic activity was defined as 1 *µ*mol p-nitrophenol released per minute and measured at 405 nm. A p-nitrophenol curve was used to determine the number of p-nitrophenol hydrolyzed micromoles.

Alpha amylase (EC 3.2.1.1) was performed using starch 1% as substrate in Tris-HCl 100 mmol/L at pH 8, utilizing the dinitrosalicylic (DNS) reagent [36]. One unit of enzymatic activity was defined as 1 *µ*mol of glucose released per minute and measured at 540 nm. A glucose curve was used to determine the number of glucose hydrolyzed micromoles. The Bradford [37] technique was used to determine the soluble protein concentration in the samples using bovine serum albumin as a standard. All enzymatic measurements were performed at 37°C and made in triplicate. Calculation of the specific activity of the extracts was performed according to Nolasco-Soria [31].

### 2.4. Statistical Analysis

Data are presented as the mean ± standard error of the mean (SE) and standard deviation (SD). Statistical analyses and graphical visualizations of the enzymatic activity were conducted in R software version 4.2.2 [38]. A linear model was fitted, and data of digestive enzyme activities were modeled as a function of DAH. The Pearson residuals were plotted against each explanatory variable, and observed data versus fitted values were used to validate this model. A *p* value < 0.05 was considered to be significant. When significant differences were observed, post hoc tests were performed using Tukey's adjustment from the Emmeans package [39]. Additionally, the patterns of variation in all the digestive enzymes for each DAH were evaluated using a multivariate analysis of variances (ANOVA) based on distances and permutations. Before the analysis, the data were scaled to zero mean and unit variance. The Euclidean distance between each pair of replicates was calculated. The total variation in this distance matrix was partitioned using a permutational multivariate analysis of variance (PERMANOVA) [40]. The null hypothesis for the DAH term in the model was created with 9,999 permutations of residuals under the reduced model. A matrix of centroids was calculated and projected in an ordination using the first two components of the principal component analysis (PCA) to visualize the pattern of similarity among DAH. Multivariate analyses were performed using the software PRIMER v7 and PERMANOVA [41].

## 3. Results

### 3.1. Larval Growth and Survival Rate

**Figure 2:**
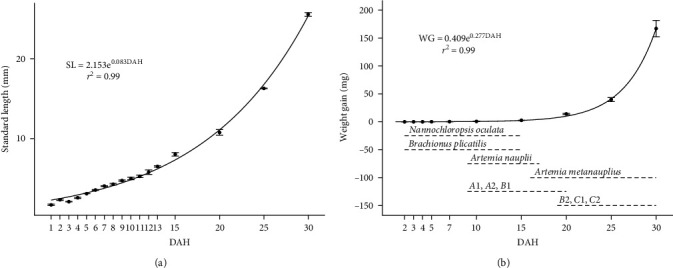
(a and b) Larval growth of the spotted seatrout (*C. nebulosus*) in terms of (a) standard length (mm) and (b) weight (mg) and feeding schedule with food type during the first 30 days after hatching (DAH). The results are expressed as mean ± standard error (SE) (*n* = 3).

The spotted seatrout larvae showed a standard length (SL) of 2.39 ± 0.06 mm (mean ± standard error (SE)) and a weight of 0.016 ± 0.00 mg at first feeding (86.7 CTU; 2 DAH); after 30 DAH (895.9 CTU), they reached a SL of 25.90 ± 0.37 mm and a weight of 167.04 ± 2.52 mg (Figure 2). During larval development, growth in SL and weight followed an exponential curve according to the following equations: SL = 2.153e^0.083DAH^ (*r* = 0.99; *n* = 3) and weight = 0.409e^0.277DAH^ (*r* = 0.99; *n* = 3). Survival at the end of the rearing period was 7.8%.

### 3.2. Digestive Enzyme Activities

**Figure 3:**
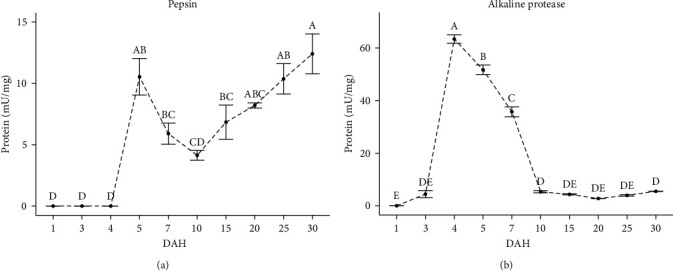
(a and b) Specific digestive enzyme activity (mU/mg protein) during larval development of spotted seatrout (*C. nebulosus*); superscript letters indicate statistical differences among ages. The results are expressed as mean ± standard error (SE) (*n* = 3 pooled samples).

The specific activities of pepsin and alkaline protease are shown in Figure 3. The specific activity of pepsin was first detected at high levels at 5 DAH (144.5 UTC; 10.56 ± 1.48 mU/mg protein) and decreased at 10 DAH (289 CTU; 4.11 ± 0.84 mU/mg protein) (*p* < 0.05); then, its activity gradually increased until 30 DAH (867 UTC; 12.4 ± 1.62 mU/mg protein) (Figure 3(a)). Specific alkaline protease activity was detected at low levels at 3 DAH (86.7 UTC; 4.43 ± 1.30 mU/mg protein) at the beginning of exogenous feeding and increased abruptly at 4 DAH (115.6 UTC; 63.41 ± 1.60 mU/mg protein) (*p* < 0.05), following a progressive decrease until 10 DAH (289 UTC; 5.33 ± 0.43 mU/mg protein) (*p* > 0.05), to remain constant until the end of the study (Figure 3(b)).

**Figure 4:**
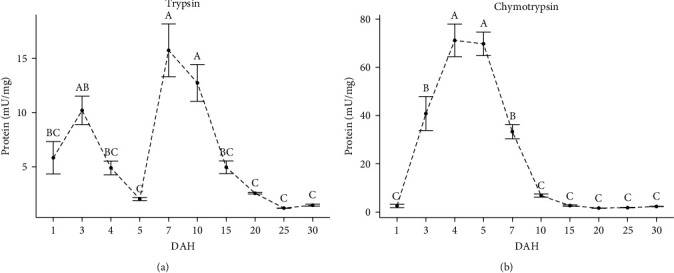
(a and b) Specific digestive enzyme activity (mU/mg protein) during larval development of the spotted seatrout (*C. nebulosus*); superscript letters indicate statistical differences among ages. The results are expressed as mean ± standard error (SE) (*n* = 3 pooled samples).

Changes in the specific activities of trypsin and chymotrypsin are shown in Figure 4. The specific activity of trypsin was detected at hatching (28.9 UTC; 5.81 ± 1.48 60 mU/mg protein). At 3 (86.7 UTC; 10.20 ± 1.29 mU/mg protein) and 7 DHA (202.3 UTC; 15.74 ± 2.41 mU/mg protein) (*p* < 0.05), trypsin showed maximum peaks in its specific activity. After 7 DAH, a decrease in specific activity was observed at 25 (722.5 UTC) and 30 DAH (867 UTC) with 1.19 ± 0.02 and 1.48 ± 0.08 mU/mg protein, respectively (*p* < 0.05). Chymotrypsin-specific activity started with low levels from hatching (28.9 UTC; 2.52 ± 0.69 mU/mg protein), and its activity increased 28-fold at 4 DAH (115.6 UTC; 71.17 ± 6.73 mU/mg protein) (*p* < 0.05) but abruptly deceased at 15 DAH (433.5 UTC; 2.69 ± 0.29 mU/mg protein) to remain constant until the end of the study (*p* > 0.05).

**Figure 5:**
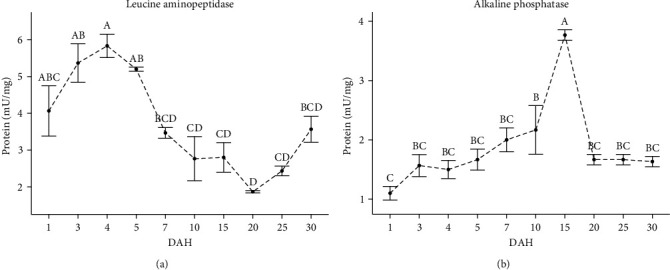
(a and b) Specific digestive enzyme activity (mU/mg protein) during larval development of the spotted seatrout (*C. nebulosus*); superscript letters indicate statistical differences among ages. The results are expressed as mean ± standard error (SE) (*n* = 3 pooled samples).

The specific activities of leucine aminopeptidase and alkaline phosphatase are shown in Figure 5. The specific activity of leucine aminopeptidase was detected from hatching (28.9 UTC; 4.05 ± 0.69 mU/mg protein) and increased until 4 DAH (115.6 UTC; 5.82 ± 0.30 mU/mg protein) (*p* < 0.05); then, its activity gradually decreased until 20 DAH (578 UTC; 1.85 ± 0.02 mU/mg protein). Alkaline phosphatase-specific activity was detected at low levels at hatching (28.9 UTC; 1.12 ± 0.10 mU/mg protein) and increased constant until 15 DAH (867 UTC; 3.78 ± 0.08 mU/mg protein) (*p* < 0.05) and then decreased abruptly to remain stable until the end of culture (1.62 ± 0.09 mU/mg protein).

**Figure 6:**
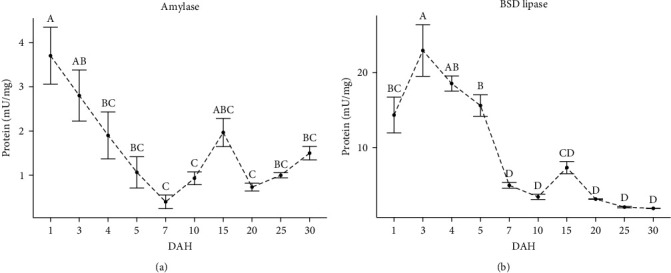
(a and b) Specific digestive enzyme activity (mU/mg protein) during larval development of the spotted seatrout (*C. nebulosus*); superscript letters indicate statistical differences among ages. The results are expressed as mean ± standard error (SE); (*n* = 3 pooled samples).

The specific activities of amylase and bile salt-dependent (BSD) lipase are shown in Figure 6. The specific activity of amylase was the highest at hatching (28.9 UTC; 3.71 ± 0.63 mU/mg protein) (*p* < 0.05), but its activity gradually decreased until 7 DAH (231.2 UTC; 0.42 ± 0.48 mU/mg protein), and then two light increases were observed at 15 (462.4 UTC; 1.94 ± 0.98 mU/mg protein) and 30 DAH (895.9 UTC; 1.50 ± 0.41 mU/mg protein) (*p* > 0.05). BSD lipase-specific activity was detected at hatching (28.9 UTC; 14.29 ± 2.37 mU/mg protein) and then reached its maximum specific activity at 3 DAH (22.95 ± 3.44 mU/mg protein) (*p* < 0.05) but gradually decreased at 7 DAH (202.3 UTC; 4.95 ± 0.37 mU/mg protein) to remain relatively constant until the end of the study (*p* > 0.05).

**Figure 7:**
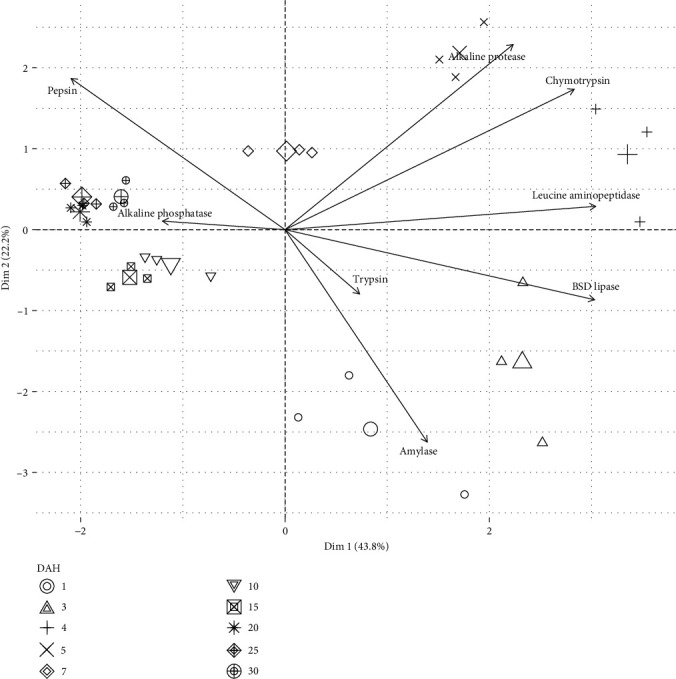
Principal component analysis (PCA) of specific digestive enzyme activity (mU/mg protein) during larval development of the spotted seatrout (*C. nebulosus*).

**Figure 8:**
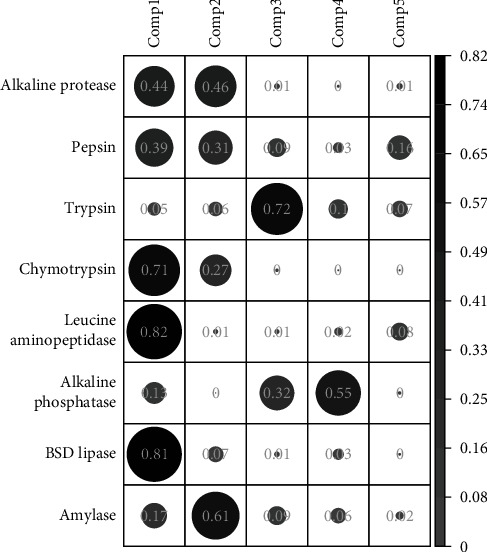
Contribution of each variable to the component.

Figure 7 shows the principal component analysis (PCA) graph of specific digestive enzyme activity. Dim 1 explained 43.8% of the variability, Dim 2 explained 22.8%, and both dimensions explained 69.6% of total variations. BSD lipase and leucine aminopeptidase enzymes contributed the most variation to Dim 1. In contrast, alkaline phosphatase and amylase enzymes contributed the most variation to Dim 2 (Figure 8). The PERMANOVA analysis found significant differences in the specific activity of digestive enzymes through time (DAH) in spotted seatrout larvae (PERMANOVA, *p*=0.0001) (Table S1). In addition, a posteriori pairwise PERMANOVA analysis detected no differences between 1 and 3 DAH (PERMANOVA, *p*=0.0577) and 25 (751.4 UTC) and 30 DAH (PERMANOVA, *p*=0.1075); all other groups were different from each other (Table S2).

## 4. Discussion

This study measured the activity of major digestive enzymes from the stomach, pancreas, and intestine during larval development to provide a basic understanding of the digestive physiology (capabilities and limitations) of the early ontogeny of *C. nebulosus*.

### 4.1. Larval Growth and Survival Rate

The growth of marine tropical fish larvae is characterized by an exponential pattern that starts slowly, followed by a rapid increase [7, 42, 43]. Exponential growth in terms of weight gained (WG) and size (SL) was observed for the spotted seatrout. Still, interestingly, this species showed faster growth compared with other tropical fish larvae, such as *Centropomus undecimalis* [10], *Lutjanus guttatus* [44], and *Totoaba macdonaldi* [45]. The survival in the present study (7.8%) was lower than that reported by [46] (30%), both studies at 30 DAH. However, the stocking density in this study was 93 larvae/L, while [46] used a stock of 1–2 larvae/L. In this study, the high mortality of spotted seatrout larvae could be attributed to cannibalism and aggression during rearing. High stocking densities promote cannibalism and aggressive behavior in the larvae of this species [47].

### 4.2. Ontogeny of Digestive Enzymes

Pepsin is a determinant enzyme in the digestion and assimilation of proteins in fish (altricial and precocial). Pepsin activity in fish larvae is associated with a functional stomach (gastric glands), which may take days or months [3]. In the spotted seatrout, the presence of pepsin activity was detected at 5 DAH (144.5 UTC). This aspect could suggest a functional stomach in the spotted seatrout larvae at 5 DAH. An early functional stomach was observed in *Atractosteus tropicus* (145 UTC) by Frías-Quintana et al. [9]. On the other hand, the levels of pepsin activity recorded at 5 DAH were similar to those recorded at the end of the larval period (Figure 3(a)). However, this is not consistent with the low degree of maturation of the stomach. Elevated levels of pepsin enzyme activity have also been observed in other fish species during the early stages of larval development [10, 48]. Elevated pepsin activity values during the early stages of larval development in fish may be overestimated due to the hydrolysis of cathepsins (intracellular acid hydrolases) [48]. Cathepsins are important enzymes in protein degradation during embryonic and early larval stages (eleutheroembryo) [49, 50], but in this study, acid hydrolysis was not detected before 5 DAH. Further research (molecular and histological tools) is needed to elucidate the mechanisms of stomach development in the spotted seatrout larvae.

During the first days of life of fish larvae (altricial and precocial), protein digestion in the digestive tract occurs in an alkaline environment by proteases of the pancreas and intestine (alkaline protease) until the development of a functional stomach (an acidic environment) [3]. In the spotted seatrout larvae, alkaline protease activity was detected at the onset of feeding (3 DAH), increased abruptly at 4 DAH, and gradually decreased until 7 DAH. The increase and decrease in alkaline protease activity during larvae transformation are considered physiological responses that are genetically preprogramed [51]. The increase in alkaline protease after the onset of feeding is associated with a compensatory mechanism to maximize protein digestion to compensate for the lack of acid digestion, while the decrease is associated with a functional stomach [7, 9, 48]. In the present study, the decline in alkaline protease activity coincides with the first pepsin activity detection, supporting the hypothesis of an early functional stomach in the spotted seatrout larvae.

Trypsin and chymotrypsin are the major or unique alkaline digestive proteases in the intestine of many fish, particularly in the larval stage due to the lack of a functional stomach [3]. In this sense, trypsin and chymotrypsin activities were detected in the spotted seatrout larvae at hatching (28.9 UTC) before opening the mouth; this finding is consistent with the report for *S. ocellata* larvae (∼27.3 UTC) [52, 53]. The presence of trypsin and chymotrypsin activity in fish embryonic and eleuteroembryonic stages is associated with the cleavage of yolk proteins [54, 55]. The profile observed for chymotrypsin activity coincides with that obtained for alkaline proteases; in contrast, trypsin activity decreased sharply at 4–5 DAH, indicating that chymotrypsin was responsible for most of the total alkaline protease activity during this period. The diet has been shown to modulate the plateau levels of some pancreatic and intestinal proteases but not the genetically programed timing of their rise or fall [51]. The decrease of trypsin activity a few days after hatching is a typical pattern in marine fish larvae [6, 10, 12, 54], which occurs regardless of feeding or starving conditions [56], live or inert food [52], and prey density [57, 58]. This decrease in trypsin levels may be associated with a programed physiological event in the development of marine fish larvae. Due to their essential role in protein digestion, trypsin and chymotrypsin are considered good indicators for assessing the nutritional status of fish larvae [59]. Trypsin has been reported to be sensitive under conditions that favor growth, while chymotrypsin plays an important role when growth is restricted or repressed [60, 61]. When trypsin activity decreased at 4–5 DAH, chymotrypsin showed the highest activity values, indicating a critical physiological phase for the spotted seatrout larvae.

In fish, developing a fully functional intestine involves the complete differentiation of enterocytes, achieved by establishing the brush border membrane [3]. During larval to juvenile transformation, digestion moves from an intracellular to a luminal location in the intestine [51]. Leucine aminopeptidase and alkaline phosphatase are enzymes in the cytosol and brush border membrane, respectively, commonly used as enterocyte cytodifferentiation markers [29]. In the present study, higher leucine aminopeptidase activity was observed in the early days of spotted seatrout larvae, indicating high intracellular digestion. This digestion is an expected response given the poor digestive tract development of the larvae a few days after hatching [9, 11, 62] (e.g., absorption and transport of nutrients). The increase in alkaline phosphatase activity coincided with a decrease in leucine aminopeptidase activity at least up to 15 DAH, indicating a change in digestion mode from intracellular to luminal location in the intestine.

In addition, it is interesting to note that alkaline phosphatase activity in the spotted seatrout declined sharply at 20 DAH. A decrease in the activity of this enzyme at the end of the larval stage has been reported in other fish species, such as *P. californicus* [6, 63], *P. maculatofasciatus* [7], *C. undecimalis* [10], and *C. nigrescens* [43]. In fish, intestinal alkaline phosphatase controls nutrient absorption and prevents the intestinal microbiota's inflammatory responses [64]. Alkaline phosphatase can reduce the toxicity of membrane-associated lipopolysaccharides of Gram-negative bacteria by removing their phosphate groups [65]. The resident microbiota also occurs during the development of the digestive tract in fish larvae [66]. In zebrafish, alkaline phosphatase has been suggested to be upregulated by the microbiota during gut colonization, preventing an inflammatory response [67]. The decrease in alkaline phosphatase activity at the end of the spotted seal larval period may be related to a physiological mechanism in response to the establishment of the resident gut microbiota.

Amylase is a pancreatic enzyme that catalyzes the endohydrolysis of *α*-1,4 glycosidic linkages of polysaccharides, such as starch and glycogen. In the spotted seatrout, amylase activity was higher at hatching and decreased steadily until 7 DAH (202.3 UTC). This pattern of amylase activity has been reported for other fish larvae [28, 44, 68]. High amylase levels during the early development of fish larvae have been suggested to be a genetically programed event associated with a natural predisposition to digest carbohydrates [69]. This limitation should not be surprising; although carbohydrates are cataloged as non-essential to fish nutrition, they can be metabolized and utilized nutritionally [70]. In the present study, amylase was found to be significantly around 1 and 3 DAH, highlighting the importance of carbohydrates in the diet of the spotted seatrout at the onset of feeding (Figure 6). In *S. ocellata*, a second increase in amylase activity was observed at 14 DAH (386.4 UCT) [52]. A modulatory effect of dietary carbohydrates on amylase activity has been observed in this species between 11 and 24 DAH [71]. Differences in the pattern of amylase activity between spotted and *S. ocellata* could be explained by changes in diet composition; however, interspecific variation in the developmental process of this enzyme cannot be excluded.

Bile salt-dependent lipase, a pancreatic enzyme with broad substrate specificity (e.g., cholesteryl esters, fat-soluble vitamin esters, tri- and monoglycerides) [72], is considered the major lipolytic enzyme in teleost fishes [73]. The present study detected BSD lipase activity at hatching when the spotted seatrout larvae depended exclusively on yolk reserve. Lazo et al. [52] found similar results for *S. ocellata* larvae. Lipolytic activity during the yolk sac larvae stage in fish is associated with lipid catabolism for energetic purposes [74]. This approach aligns with the reported changes in lipid composition found in the yolk reserve during this stage of the fish [75, 76]. The spotted seatrout showed the highest levels of BSD lipase activity at the onset of feeding and then decreased continuously until 7 DAH. The development of BSD lipase along the fish larvae transformation is highly variable among species [43, 52, 55]. The lipid content of the diet or live food has been observed not to have a modulatory effect on the development of BSD lipase during fish larval transformation [71, 77]. Therefore, the development of BSD lipase may follow an ontogenetic programing pattern [74]. Lipids are a source of energy, but they also provide essential fatty acids for cell structure and essential compounds [73]. In fish larvae, the onset of feeding is often followed by an exponential growth phase (intensification in metabolic processes), as observed for this species. The high lipolytic activity found in the spotted seatrout larvae at the beginning of feeding compared to later days suggests a high demand for lipids at this stage of development.

During the transformation of the spotted seatrout from larvae to juvenile, three development stages of digestive enzymes have been identified. The first stage occurred between 1 and 3 DAH, with amylase and BSD lipase being the main digestive enzymes. This period of digestive stability suggests that carbohydrates and lipids are significant components of the early diet of the spotted seatrout. The second stage occurred from 4 to 20 DAH; leucine aminopeptidase, chymotrypsin, and alkaline proteases were identified as the predominant enzymes at 4–5 DAH. At the beginning of this phase, an accelerated shift was observed towards protein digestion, indicating a high demand, which is consistent with the high growth rate during this stage. In addition, the lack of similarity between treatments means constant and accelerated physiological changes in the digestive tract of the spotted seatrout larvae. The third stage occurred between 25 and 30 DAH, where pepsin was the main enzyme. This aspect is a period of digestive stability that can be interpreted as the digestive tract maturation and transformation process completion. In addition, the fact that pepsin was the enzyme of higher activity during this period suggests that the spotted seatrout is highly protein-dependent, a typical characteristic of carnivorous fish species.

## 5. Conclusion

In the present study, spotted seatrout larvae showed an enzymatic profile typical of carnivorous species. Furthermore, pepsin activity at 5 DAH (144.5 UTC) suggests an early functional stomach; however, the biochemical activity of this enzyme needs to be complemented by molecular and histological techniques (presence of gastric cells). The period of digestive stability recorded between 25 (751.4 UTC) and 30 (895.9 UTC) DAH indicates that the maturation of the digestive system and the completion of the transformation process in spotted seatrout occur at 25 DAH. A holistic understanding of their digestive physiology is essential for determining an optimal weaning period for spotted seatrout larvae. Therefore, it is recommended to conduct studies on the RNAm expression of digestive enzymes, the gut microbiota, and the histological development of the digestive tract. Furthermore, the digestion pattern obtained here should be considered when designing compound diets for weaning the spotted seatrout.

## Figures and Tables

**Table 1:** Feeding schedule and food types during larval rearing of the spotted seatrout (*C. nebulosus*).

Food item	Type	Density	Period (DAH)
Microalgae	*Nanocloropsis oculata*	0.5–10 ×10^6^ cells/mL	1–15
Enriched rotifer	*B. rotundiformis*	7–17 rotifer/mL	1–15
*Artemia* nauplii	*A. franciscana*	1–2 nauplii/mL	9–17
Enriched *Artemia* metanauplii	*A. franciscana*	2–4 metanauplii/mL	16–30
Otohime™	A1 (75–150 *µ*m), A2 (150–250 *µ*m), B1 (250–360 *µ*m), B2 (360–650 *µ*m), C1 (580–840 *µ*m) and C2 (840–1,410 *µ*m)	Ad libitum	9–12, 11−16, 15–20, 19−24, 23–28, 27−30

## Data Availability

The data that support the findings of this study are available from the lead author.

## Supplementary Materials

Supplementary Materials Permutational multivariate analysis of variance (PERMANOVA) information.
