# Supplementary material for: Changes in Digestive Enzyme Activities during Larval Development of Spotted Seatrout (Cynoscion nebulosus)
Source: Aquac Nutr. 2024 May 8;2024:1309390. doi: 10.1155/2024/1309390 (PMC11095990; doi:10.1155/2024/1309390)
Supplement: Supplementary Materials — Permutational multivariate analysis of variance (PERMANOVA) information. [file 1309390.f1.docx]

**Supplementary Information**

Table 1. Results of the PERMANOVA test performed on the activity of digestive enzymes in spotted seatrout larvae. DHA = days after hatching; Df = degrees of freedom; SS = sum of squares; MS = mean square; Perm = permutations.

| **Source** | **Df** | **SS** | **MS** | **Pseudo-F** | **P (perm)** |
| --- | --- | --- | --- | --- | --- |
| DAH | 9 | 208.66 | 23.184 | 19.864 | 0.0001 |
| Residual | 20 | 23.343 | 1.1671 |  |  |
| Total | 29 | 232 |  |  |  |

Table 2. *A posteriori* pair-wise permutational multivariate analysis of variance comparison for the activity of digestive enzymes in the spotted seatrout larvae.

| **Groups** | **T** | **P(perm)** | **Groups** | ***T*** | **P(perm)** |
| --- | --- | --- | --- | --- | --- |
| **1 vs 3** | 1.9101 | 0.0577 | **4 vs 30** | 6.7127 | 0.0004 |
| **1 vs 4** | 3.994 | 0.0039 | **5 vs 7** | 4.4426 | 0.0023 |
| **1 vs 5** | 4.5842 | 0.0016 | **5 vs 10** | 4.7715 | 0.0014 |
| **1 vs 7** | 4.2247 | 0.0036 | **5 vs 15** | 6.1694 | 0.001 |
| **1 vs 10** | 3.1441 | 0.0075 | **5 vs 20** | 7.2783 | 0.0006 |
| **1 vs 15** | 4.2138 | 0.0037 | **5 vs 25** | 6.2906 | 0.0006 |
| **1 vs 20** | 4.4048 | 0.0061 | **5 vs 30** | 5.1898 | 0.0018 |
| **1 vs 25** | 4.2523 | 0.0042 | **7 vs 10** | 1.9413 | 0.0439 |
| **1 vs 30** | 3.8586 | 0.0065 | **7 vs 15** | 4.7862 | 0.0024 |
| **3 vs 4** | 2.9338 | 0.0123 | **7 vs 20** | 5.3502 | 0.0033 |
| **3 vs 5** | 3.8172 | 0.0053 | **7 vs 25** | 5.2227 | 0.004 |
| **3 vs 7** | 3.795 | 0.0039 | **7 vs 30** | 4.8265 | 0.0028 |
| **3 vs 10** | 3.3584 | 0.0057 | **10 vs 15** | 3.0724 | 0.0109 |
| **3 vs 15** | 4.5156 | 0.0016 | **10 vs 20** | 2.9787 | 0.0174 |
| **3 vs 20** | 5.3539 | 0.0012 | **10 vs 25** | 3.2377 | 0.0084 |
| **3 vs 25** | 5.1998 | 0.0012 | **10 vs 30** | 3.3283 | 0.0084 |
| **3 vs 30** | 4.7 | 0.0018 | **15 vs 20** | 5.709 | 0.0015 |
| **4 vs 5** | 2.9943 | 0.0094 | **15 vs 25** | 5.1747 | 0.0015 |
| **4 vs 7** | 4.7311 | 0.0016 | **15 vs 30** | 4.7123 | 0.0026 |
| **4 vs 10** | 5.0076 | 0.0019 | **20 vs 25** | 2.0631 | 0.052 |
| **4 vs 15** | 6.7265 | 0.0007 | **20 vs 30** | 3.4097 | 0.0161 |
| **4 vs 20** | 8.4042 | 0.0003 | **25 vs 30** | 1.791 | 0.1075 |
| **4 vs 25** | 7.7127 | 0.0003 |  |  |  |
